# Psychometric properties of an Arabic translation of the short form of the affective lability scale in a sample of Lebanese adults

**DOI:** 10.3389/fpsyg.2026.1642617

**Published:** 2026-01-23

**Authors:** Gaelle Kanj, Diana Malaeb, Fouad Sakr, Mariam Dabbous, Sahar Obeid, Souheil Hallit, Feten Fekih-Romdhane

**Affiliations:** 1 School of Arts and Sciences, Holy Spirit University of Kaslik, Jounieh, Lebanon; 2 College of Pharmacy, Gulf Medical University, Ajman, United Arab Emirates; 3 School of Pharmacy, Lebanese International University, Beirut, Lebanon; 4 Department of Psychology and Education, School of Arts and Sciences, Lebanese American University, Jbeil, Lebanon; 5 School of Medicine and Medical Sciences, Holy Spirit University of Kaslik, Jounieh, Lebanon; 6 Applied Science Research Center, Applied Science Private University, Amman, Jordan; 7 Department of Psychiatry “Ibn Omrane”, Razi Hospital, The Tunisian Center of Early Intervention in Psychosis, Manouba, Tunisia; 8 Faculty of Medicine of Tunis, Tunis El Manar University, Tunis, Tunisia

**Keywords:** affective lability, anxiety, Arabic, depression, psychometric properties, self-esteem, stress

## Abstract

**Background:**

The present study aimed to investigate the psychometric properties of an Arabic version of the Affective Lability Scale in its short form (ALS-18) within an Arabic-speaking sample. Particularly, the concurrent validity, sex invariance and factorial structure were examined.

**Methods:**

The total sample of this cross-sectional study consisted of 748 adults, with a mean age of 34.48 ± 13.25 years, 66.5% females. After completing the forward-backward translation for cultural and linguistic adaptation, concurrent validity was assessed through correlations with related constructs, Confirmatory Factor Analysis was conducted to examine the factorial structure, and internal reliability as well as measurement invariance across sex were tested, the latter being through multigroup analyses.

**Results:**

The fit of the scale’s original three-factor model was suggested through confirmatory factor analyses. Full measurement invariance at the configural, metric, and scalar levels was attained. The scale also yielded concurrent validity, with results indicating associations with increased levels of depression, anxiety and stress, and lower levels of self-esteem. The study’s findings further denoted good internal consistency of the Arabic ALS-18 with values of McDonald’s *ω* and Cronbach’s *α* greater than 0.70.

**Conclusion:**

Results revealed that the Arabic ALS-18 is a reliable and valid self-report measure that could be utilized among an Arabic-speaking population to assess affective lability. The availability of the Arabic version of the ALS-18 is deemed to increase its use for research and provide a foundation for future clinical validation studies, globally benefiting Arabic-speaking individuals.

## 1 Introduction

Affective Lability (AL) refers to rapid, frequent, and intense fluctuations in expression of emotions, leading to challenges in regulating these shifts and their behavioral effects (Bandyopadhyay Prasanta et al., 2014; Leaberry et al., 2020). Referring to a type of emotional reactivity, AL can incite heightened emotional reactions among individuals when faced with life events reminiscent to past emotional experiences that had a significant impact on them (Almeida et al., 2024). Harvey et al. (1989) describe AL as the tendency to shift between various mood states similar to anger, hypomania, anxiety and depression. The prevalence of AL is found among individuals with diverse mental health disorders such as personality disorders, psychotic disorders, anxiety disorders or obsessive-compulsive disorders (Bandyopadhyay Prasanta et al., 2014; Høegh et al., 2020). In such clinical populations, AL predicts multiple debilitating outcomes, such as disease progression, increased clinical burden and impaired social functioning (Høegh et al., 2022), cognitive deficits (Lee et al., 2012), as well as suicidal ideation and attempts (Wedig et al., 2012). Beyond simply being a feature of several psychiatric disorders, AL is also a difficulty which can occur throughout the lifespan, and which was reported to be highly prevalent in the adult general population (Marwaha et al., 2013). In addition, AL observed in individuals from the general population (without a diagnosed mental disorder) was suggested to be clinically significant, and likely involved in the etiology and early, undiagnosed phases of a range of mental disorders (Broome et al., 2015). Considering that AL is closely linked to the development of mental disorders, a psychometrically sound measure is required to accurately assess the construct in both clinical practice and scientific research.

One comprehensive psychometric tool designed with the aim of evaluating changes across diverse modalities of affective functioning; assessing oscillations from one’s baseline mood (euthymic) to other affective domains, is the Affective Lability Scale (ALS) (Harvey et al., 1989). The original 54-item ALS developed by Harvey et al. (1989) consists of six subscales elaborated to tap into one’s physiological perceptions, subjective experiences and behaviors, including “*depression, anxiety, elation, depression/anxiety, anger, and biphasic affect (depression/elation)*” (Oliver and Simons, 2004). These subscales are generated for six types of shifts in affect, measured through three dimensions, namely perceptions of behavioral, physiological, and emotional changes associated with cognition (Harvey et al., 1989). To address the ALS’s restricted empirical support concerning its factor structure (Aas et al., 2015) and length, a shorter version of 18 items (ALS-18) was elaborated by Oliver and Simons (2004), comprising a three-factor model of AL. In fact, the initial six conceptual scales were refined through the selection of items that contributed most to each subscale’s internal consistency (Oliver and Simons, 2004). While ensuring subscales retained at least 5 items and maintained a reliability score greater than 0.75, items were excluded following an iterative elimination process, resulting in a 33-item scale (Oliver and Simons, 2004). A factor analysis was then conducted on the latter, identifying seven eigenvalues above 1.0, whereas the scree plot suggested a three-factor solution, best categorized as Anxiety/Depression, Depression/Elation, and Anger (Oliver and Simons, 2004) The scale was further refined by iteratively excluding items based on low item-total correlations maintaining a scale alpha greater than 0.80 with at least five items for each of the three scales, resulting in the 18-item ALS (Oliver and Simons, 2004). The latter scale retains a minimum of two items from the initial ALS’s six scales and shows a strong correlation with the total score of the original ALS (*r* = 0.94) (Oliver and Simons, 2004).

The development of the ALS and ALS-18 was initially carried out in the English language and has since been adapted and/or validated in numerous other languages including Chinese (Xu et al., 2022), Greek (Kalantzi et al., 2022), and Italian (Contardi et al., 2018). The two latter studies showed good internal reliability and consistency for the entirety of the ALS-18’s factors (Kalantzi et al., 2022; Contardi et al., 2018). In fact, a minimum value of 0.85 for Cronbach’s alpha was found for all three factors. Conversely, the Chinese study (Xu et al., 2022) yielded contrary results; failing to support the three-model factor of the initial validation of the ALS-18 through its factor analysis. Nonetheless, the ALS-18 has not yet been validated in Arabic, to the extent of our knowledge. In addition, ALS is a notable aspect of affect dysregulation on which a restricted number of research has been conducted in Arab contexts (Merhi and Kazarian, 2015). The lack of validated or adapted measures across cultures might pertain to that limit in the literature. Given that affect regulation has a culturally dependent nature (Matsumoto et al., 2008), significant differences might be found between Western and Arab samples regarding AL. The validation of the ALS-18 among an Arabic-speaking sample might give rise to the appropriateness and usefulness of the scale in an Arab setting. Moreover, a shorter version, being further concise in nature, was deemed advantageous in terms of the administration of a scale examining AL among an Arab sample. In fact, the reduction in completion time, costs, and burden on respondents within Arab countries with low to middle income are noteworthy (Fekih-Romdhane et al., 2023) as it increases response rates and data quality (Franke et al., 2013). Given the aforementioned factors, the requirement for an Arabic version of the ALS-18 adequately translated and validated becomes evident. Because AL is commonly experienced in general population adults and is potentially relevant to an array of mental health outcomes, we believe that examining the psychometric properties of the ALS-18 in such a population would be an important contribution to the body of knowledge about psychometric properties of the scale.

Subsequently, the objective of the current study was to assess the psychometric properties of an Arabic Translation of the short form of the ALS (ALS-18) in a non-clinical sample of Arabic-speaking adults from Lebanon. It is hypothesized that the scale in its Arabic version will (1) yield three-factor solution consistent with the original model, (2) show good internal reliability, (3) be invariant across sex groups, and (4) have adequate concurrent and divergent validity.

## 2 Methods

### 2.1 Procedures

This study has been conducted over a period of 1 month, from August 1 to August 31, 2023 through a cross-sectional approach, suitable for psychometric validation, as it provides a broad snapshot of the scale’s performance across a large sample at a single point in time and yields the necessary data variance to test the scale’s stability without the confounding effects of time-based changes (So et al., 2025). A mixed convenience and snowball sampling strategy was adopted. The research team invited potential eligible participants to complete the survey via Google forms; those who agreed to take part in the study were then requested to share the link with others. Inclusion criteria comprised being an Arabic-speaking adult aged 18 years and over, with access to the Internet, who resides in and is a citizen of Lebanon. Exclusions encompassed individuals who self-reported a physician’s diagnosis of mental health disorders and those declining to complete the questionnaire. We restricted eligibility to Lebanese citizens to ensure a relatively homogenous sociocultural context and a stable sampling frame. In Lebanon, non-citizen residents constitute heterogenous groups (migrant workers, refugees, short-term residents) with potentially different living conditions and stress exposures that may influence affective lability and response patterns. The survey was conducted anonymously and participation was voluntary and without compensation. The study protocol was approved by the ethics committee of the School of Pharmacy at the Lebanese International University (Reference # 2023RC-023-LIUSOP). Participants were presented with an online information sheet detailing the study’s objectives, procedures, anonymity, voluntariness and data use prior to accessing the survey. Informed consent was then obtained from all subjects for study participation; the online submission of the soft copy was considered equivalent to receiving a written informed consent (Swami et al., 2022).

### 2.2 Minimum sample size calculation

Monte Carlo simulations using different seed values and factor loadings suggested that a sample size of approximately 180 persons would be adequate to have enough statistical power for the CFA (Marcoulides and Chin, 2013), a threshold exceeded in our sample.

### 2.3 Measures

#### 2.3.1 The affective lability scale (ALS-18)

The scale is composed of 18 items, rated on a 4-point Likert scale (“0 = very uncharacteristic of me” to “3 = very characteristic of me”). It yields three subscales: Anxiety/Depression, Depression/Elation, and Anger. The total score ranges between 0 and 54; higher scores indicate heightened affective lability. The forward and backward translation method was applied to the ALS-18 following international guidelines (Beaton et al., 2000). The English version was translated to Arabic by a Lebanese translator who was completely unrelated to the study. Afterwards, a Lebanese psychologist with a full working proficiency in English, translated the Arabic version back to English. The initial and translated English versions were compared to detect and later eliminate any inconsistencies by a committee composed of the research team and the two translators (Fekih-Romdhane et al., 2023; Bitar et al., 2024). To ensure comprehension and cultural appropriateness, a pilot study was conducted on 30 people before the start of the official data collection to ensure all questions are well understood; no changes were made consequently.

#### 2.3.2 The Arabic version of the depression anxiety stress scale (DASS-8)

Ali et al. (2021), a validated short-form scale, measures three constructs depression (three items, e.g., “*felt down hearted and blue*”), anxiety (three items, e.g., “*felt scared without reason*”), and stress (two items, e.g., “*was using a lot of my mental energy*”) through eight items rated on a four-point Likert scale. Higher scores indicate higher depression, anxiety and stress, respectively (Ali et al., 2024). The reliability of the scale was good as follows: Depression (*ω* = 0.85/*α* = 0.85), Anxiety (*ω* = 0.85/*α* = 0.85), and Stress (*α* = 0.77).

#### 2.3.3 The Arabic version of the single item self-esteem scale (A-SISE)

This is a validated Arabic adaptation of the original scale, consists of a single item in English as “*Please indicate to what extent the following statement applies to you. I have high self-esteem*,” and designed to efficiently assess global self-esteem (Brailovskaia and Margraf, 2020). Respondents are asked to rate the item on a 5-point Likert Scale (“*1 = not at all true of me, 2 = rather not true of me, 3 = some part true of me, 4 = rather true of me, 5 = very true of me*”) (Fekih-Romdhane et al., 2023).

#### 2.3.4 Demographics

Participants were asked to provide their demographic details consisting of age, sex, marital status, education level, place of living, and household density.

### 2.4 Analytic strategy

#### 2.4.1 Confirmatory factor analysis (CFA)

There were no missing responses in the dataset. Data from the total sample was used to conduct a CFA using the SPSS AMOS v.29 software. Therefore, a minimum sample of 360 participants was assumed needed to have enough statistical power based on a ratio of 20 participants per one item of the scale, which was exceeded in our sample. Our intention was to test the original model of the ALS-18 scores (i.e., three-factor model (Look et al., 2010)) and, if divergent, carry out an exploratory-to-confirmatory factor analysis. Parameter estimates were obtained using the maximum likelihood method and fit indices. To check if the model was adequate, several fit indices were calculated: the normed model chi-square (*χ*^2^/df), the root mean square error of approximation (RMSEA), standardized root mean square residual (SRMR), the Tucker-Lewis Index (TLI) and the comparative fit index (CFI). Values ≤ 5 for *χ*^2^/df, and ≤0.08 for RMSEA, and 0.95 for CFI and TLI (Hu and Bentler, 1999), and ≤0.05 for SRMR (Byrne, 2013) indicate good fit of the model to the data. Additionally, the Akaike Information Criterion (AIC) and Bayesian Information Criterion (BIC) were calculated for model comparisons. A difference <2 between AIC and BIC values indicated that both models were acceptable, whereas a difference >10 favored the model with the lower value (Burnham and Anderson, 2004). Multivariate normality was not verified at first (Bollen-Stine bootstrap *p* = 0.002); therefore, non-parametric bootstrapping procedure was performed. Evidence of convergent validity was assessed in this sample using the average variance extracted (AVE), with values of ≥ 0.50 considered adequate (Malhotra, 2020).

#### 2.4.2 Sex invariance

To examine sex invariance of ALS-18 scores, multi-group CFA (Chen, 2007) using the total sample was conducted. Measurement invariance was assessed at the configural, metric, and scalar levels (Vandenberg and Lance, 2000). ΔCFI ≤ 0.010 and ΔRMSEA ≤ 0.015 or ΔSRMR ≤ 0.010 (0.030 for factorial invariance) were accepted as evidence of invariance (Chen, 2007).

### 2.5 Reliability and validity analyses

Internal reliability was assessed using McDonald’s *ω* and Cronbach’s *α*, with values greater than 0.70 reflecting adequate reliability (Dunn et al., 2014). All ALS-18 subscales scores were considered normally distributed according to their skewness and kurtosis values varying between ±1 (Hair et al., 2023). Consequently, the Pearson test was used to correlate those scores with DASS-8 and A-SISE subscales scores. The independent samples *t* test was used to compare the ALS-18 subscales scores between sex groups. *p* < 0.05 was considered statistically significant.

## 3 Results

### 3.1 Sociodemographic characteristics of the participants

A total of 748 adults filled the survey, with a mean age of 34.70 ± 13.26 years [min = 18; max = 72] and 65.9% females. All participants’ details can be found in Table 1.

**Table 1:** Sociodemographic characteristics of the participants (*n* = 680).

Variable	Mean ± SD or *n* (%)
Age (years)	34.70 ± 13.26
Household crowding index (person/room)	1.20 ± 0.61
Sex	
Male	232 (34.1%)
Female	448 (65.9%)
Marital status	
Single	318 (46.8%)
Married	329 (48.4%)
Divorced	18 (2.6%)
Widowed	15 (2.2%)
Education	
Primary	17 (2.5%)
Complementary	72 (10.6%)
Secondary	115 (16.9%)
University	476 (70.0%)
Place of living	
Urban	327 (48.1%)
Rural	353 (51.9%)

### 3.2 Confirmatory factor analysis of the ALS-18

CFA indicated that fit of the three-factor model of the ALS-18 scale was acceptable: *χ*^2^/df = 482.36/132 = 3.65, RMSEA = 0.063 (90% CI 0.057, 0.069), SRMR = 0.027, CFI = 0.963, TLI = 0.957. The standardized estimates of factor loadings were all adequate for all models. The AVE value was also adequate for the total scale (=0.63), depression-anxiety factor (=0.63), depression-elation factor (=0.62) and anger factor (=0.64). The reliability of the scale was good as follows: total score (*ω* = 0.96/*α* = 0.96), Anxiety/Depression (*ω* = 0.89/*α* = 0.89), Depression/Elation (*ω* = 0.93/*α* = 0.93), and Anger (*ω* = 0.90/*α* = 0.90) respectively.

**Figure 1:**
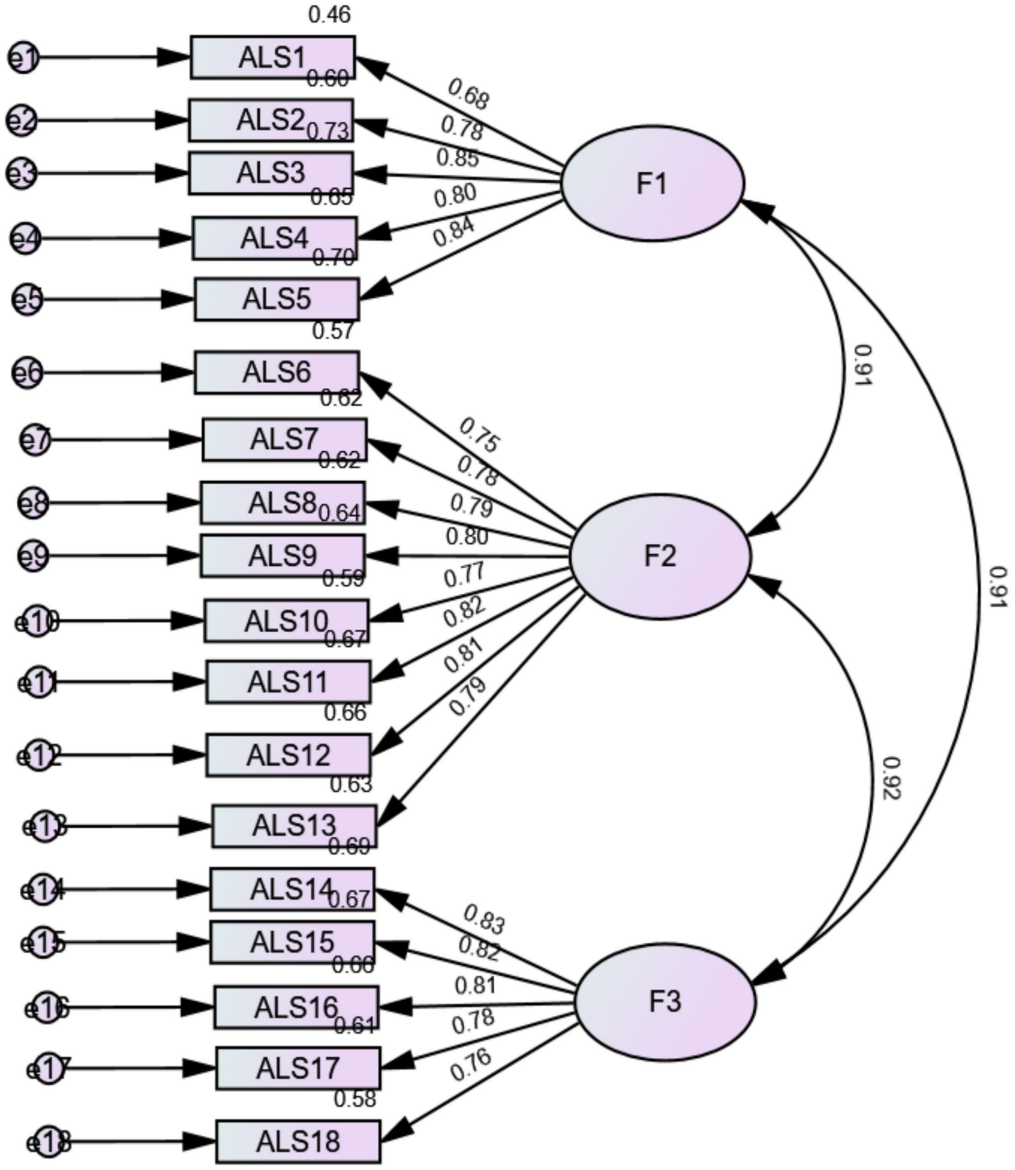
Factor loadings derived from the confirmatory factor analysis of the three-factor model of the affective liability scale in the total sample. F1, Depression-Anxiety; F2, Depression-Elation; F3, Anger.

CFA of the second-order model indicated that fit of the total ALS-18 score was acceptable: *χ*^2^/df = 482.36/132 = 3.65, RMSEA = 0.063 (90% CI 0.057, 0.069), SRMR = 0.027, CFI = 0.963, TLI = 0.957 (Figure 1).

### 3.3 Sex invariance of the ALS-18 scale

We were able to show the invariance across sex at the configural, metric, and scalar levels (Table 2). Females had significantly higher depression-anxiety (6.12 ± 3.57 vs. 5.33 ± 3.60, *t*(678) = −2.73, *p* = 0.007, Cohen’s *d* = 0.220), depression-elation (10.40 ± 5.63 vs. 8.77 ± 5.63, *t*(678) = −3.57, *p* < 0.001, Cohen’s *d* = 0.289), anger (6.03 ± 3.65 vs. 5.31 ± 3.67, *t*(678) = −2.42, *p* = 0.016, Cohen’s *d* = 0.196) and ALS total scores (22.54 ± 12.10 vs. 19.41 ± 12.24, *t*(678) = −3.19, *p* = 0.002, Cohen’s *d* = 0.258).

**Table 2:** Measurement invariance of the affective liability scale across sex in the total sample.

Model	CFI	RMSEA	SRMR	Model comparison	ΔCFI	ΔRMSEA	ΔSRMR
Configural	0.941	0.056	0.043				
Metric	0.940	0.055	0.046	Configural vs. metric	0.001	0.001	0.003
Scalar	0.940	0.054	0.046	Metric vs. scalar	<0.001	0.001	<0.001

CFI, Comparative fit index; RMSEA, root mean square error of approximation; SRMR, Standardized root mean square residual.

### 3.4 Convergent-divergent validity of the ALS-18 scale

Higher ALS-18 total, ALS-18 depression-anxiety, ALS-18 depression-elation and ALS-18 anger scores were significantly and moderately associated with higher depression, anxiety and stress (as measured by DASS-8 scale) and weakly associated with lower self-esteem (Table 3).

**Table 3:** Correlation matrix of continuous variables.

	1	2	3	4	5	6	7
1. ALS-18 total	1						
2. ALS-18 depression-anxiety	0.93^***^	1					
3. ALS-18 depression-elation	0.96^***^	0.84^***^	1				
4. ALS-18 anger	0.93^***^	0.82^***^	0.84^***^	1			
5. DASS Depression	0.51^***^	0.49^***^	0.47^***^	0.49^***^	1		
6. DASS Anxiety	0.53^***^	0.52^***^	0.48^***^	0.53^***^	0.83^***^	1	
7. DASS stress	0.49^***^	0.47^***^	0.46^***^	0.47^***^	0.77^***^	0.73^***^	1
8. Self-esteem	−0.12^**^	−0.14^***^	−0.17^**^	−0.11^**^	−0.14^**^	−0.16^***^	−0.06

^**^p < 0.01; ^***^p < 0.001.

## 4 Discussion

AL is a broad term employed to allude to emotion fluctuations, and is considered a predictor of various mental health disorders’ features, clinical course, and outcomes, including bipolar, personality or anxiety disorders (Bandyopadhyay Prasanta et al., 2014; Strawbridge et al., 2022). For research purposes as well as preventive and clinical practice, the availability of reliable and valid scales for AL screening is of high importance. In this vein, validating the short form of the ALS in the Arabic language was essential to better assess its practicality within the settings of Arab research. Factually, the administration of short scales requires reduced time and effort from respondents, heightening its convenience among the Arab population contending with a lack of psychometric soundness in tools for research, resources’ shortage, and financial turmoil (Maalouf et al., 2019). The investigation of the psychometric properties of the Arabic ALS-18 conducted through this present study revealed a good reliability and acceptable fit of the ALS-18 three-factor model and second-order model. The latter suggests that the three first-order factors are explained by a higher-order latent factor. Given that the three-factor model already exhibits strong correlations among factors (*r* > 0.90 across all items) and shows identical fit indices to the second-order model, the latter does not significantly alter the established structure of the three-factor model but rather reinforces the notion that these factors can be integrated into a higher-order construct. Both models were then retained as (1) the three-factor model would be valuable for research studying the subdimensions independently, while (2) the second-order model provides a global ALS-18 score, making it more useful in settings where a single composite score is preferred. The three-factor model in the study conducted by Doğan and Şenormanci (2025) highlighted an acceptable fit, aligning with previous studies on the ALS-18 structure (Broome et al., 2015; Xu et al., 2022; Ali et al., 2021). Findings of Contardi et al. (2018) suggest the presence of a higher-order factor, proposing the computation of a total score, as commonly reported in the literature (Aas et al., 2015; Look et al., 2010). However, the latter research (Contardi et al., 2018) was not conclusive in determining the superiority of the three-factor or hierarchical model. Correlations between the latent factors were high, suggesting substantial shared variance among the affective lability dimensions. This pattern is theoretically consistent with the conceptualization of affective lability as a higher-order construct and provides support for the plausibility of a second-order model representing a global affective lability factor. Although the *χ*^2^/df ratio was relatively elevated, this index is well known to be highly sensitive to sample size and model complexity, often leading to statistically inflated values in large samples. In contrast, the incremental fit indices (CFI and TLI), which compare the specified model to a null model and are less affected by sample size demonstrated excellent fit. Together with the low SRMR, these indices indicate that the proposed factor structure provides a robust and meaningful representation of the data despite the elevated *χ*^2^/df. Additionally, the scale showed notable concurrent validity, as well as an invariance across sex. In summary, the Arabic version of the ALS-18 is presumed suitable and convenient for screening affective lability among non-clinical Arab populations.

The internal consistency of the Arabic ALS-18 underscored in our results is deemed adequate for the scale’s three dimensions as follows: Anxiety/Depression (AD) (*ω* = 0.89/*α* = 0.89), Depression/Elation (DE) (*ω* = 0.93/*α* = 0.93), and Anger (*ω* = 0.90/*α* = 0.90). Our results are consistent with previously conducted studies investigating the psychometric properties of the 3-factor ALS-18. Among a population of undergraduates, Cronbach’s alpha coefficients obtained in the first study of the development of the ALS Short Form (Oliver and Simons, 2004) were of 0.87 for AD, 0.81 for DE, and 0.82 for Anger. An investigation of the psychometric properties carried out a few years later among individuals with personality disorders and control groups (Look et al., 2010), and within a Chinese population (Xu et al., 2022) yielded similar results, with coefficient alpha, respectively, equaling 0.82 and 0.87 for AD, 0.78 and 0.865 for DE, as well as 0.87 and 0.81 for Anger. The use of McDonald’s ω presents an advantage to the current study, in comparison with only using Cronbach’s alpha coefficient in the foregoing research, as the former coefficient’s effectiveness is considered greater than that of the latter (Crutzen, 2017).

Moreover, our findings underscore an invariance across sex at the metric, scalar and configural levels among an Arabic-speaking sample, highlighting that its factorial structure do not vary between sex in the stated population (Fekih-Romdhane et al., 2023). To the extent of our knowledge, the measurement invariance across sex is scarce in the literature. Indeed, few studies have examined this characteristic, one of which was conducted by Harvey et al. (1989) on the ALS initial 54-item version. The later researchers highlighted differences among sex solely for the depression scale. Similarly, in their research within a Portuguese sample, Almeida et al. found notably higher scores among women than men on the total and subscales’ scores (Almeida et al., 2024). Contardi et al. (2018) contend that the ALS-18’s scores show no association with sex. The present study showed significantly higher scores on AD, DE, and Anger among female respondents in comparison to male respondents. The distinctions in emotional experiences among males and females have indeed been underscored through precedent research, particularly concerning the frequency of negative emotions (Almeida et al., 2024). In fact, Gunnlaugsson et al. (2011) postulates that women have a predisposition to heightened levels of AL. This may explain the increased susceptibility of females to diverse mental health disorders relating to emotions and its fluctuations (Staugaard and Berntsen, 2021; Bradley et al., 2001).

Findings of the present study highlighted a significant association between greater scores on all three ALS-18 dimensions (i.e., depression-anxiety, depression-elation, and anger) and higher depression, anxiety, and stress, indicating that individuals with increased AL would tend to experience high levels of psychological distress. In fact, greater ALS-18 total scores, ALS-18 depression-anxiety, depression-elation, and anger with DASS-8 Depression, Anxiety, and Stress indicate significant and moderate positive associations. These associations suggest that individuals scoring higher on affective lability would tend to report greater scores on the latter variables. Results of the ALS short form validated by Xu et al. (2022) denoted a positive correlation between affective lability, and anxiety and depression. Particularly, stress resistance and adaptability were shown to be positively impacted by the role depression-elation plays (Xu et al., 2022). In their study focused on the psychometric properties of the ALS-18. Contardi et al. (2018) highlighted a significant association between the scale’s general factors and subdimensions, and corresponding instruments assessing difficulties in emotion regulation and depression. These results would also suggest the role of a risk factor that affective lability may play for depression, anxiety, and stress (Xu et al., 2022). Despite the contrasting results from Oliver and Simons (Marwaha et al., 2013) offering no further clarifications, our findings are supported by numerous previous studies (Xu et al., 2022; Contardi et al., 2018; Weibel et al., 2019; Li, 2023). In fact, the association observed can be uncovered by the high comorbidity existing between depression and anxiety (Pollack, 2005). Furthermore, a link between depression and the ALS-18 factor of anger was established, supporting the idea that anger is a prominent feature of depression and significantly contributes to its development (Busch, 2009). Moreover, stress is identified among affective lability, depression, and anxiety (Li et al., 2021).

Additionally, an association between higher ALS depression-anxiety, ALS depression-elation and ALS anger, and lower self-esteem was denoted. Self-esteem has become central in psychology, defined as “*the individual’s positive or negative attitude toward the self as a totality*” (Rosenberg et al., 1995). It has been shown that poor self-esteem correlates with various mental disorders with affect and emotion regulation emerging as crucial factors (Mouatsou and Koutra, 2023). In other words, self-esteem is associated with the two extensively studied emotion regulation strategies: individuals employing reappraisal to manage emotions tend to exhibit higher self-esteem (Mouatsou and Koutra, 2023). Furthermore, fluctuations in self-esteem and mood are significant characteristics observed in clinical conditions like depression (Kashdan et al., 2006). Individuals with low self-esteem often interpret their daily events as less positive and more influential on their emotional states (Campbell et al., 1991). Our findings showed that, although the correlation between self-esteem and AF was statistically significant (likely due to the large sample size), the magnitude of the correlations was small (in the range of 0.03–0.15). This suggests that self-esteem and AL are not similar constructs, thus attesting for the divergent validity of the scale.

### 4.1 Limitations and research perspective

The current study has several notable strengths worth highlighting. The recruitment of an adequate sample size, the involvement from both sex groups, and the use of validated measures for concurrent validity analyses enrich the research. Furthermore, validating and investigating the psychometric characteristics of a shortened version of the ALS in an Arab-speaking community marks a significant step forward in the field. Another advantage would be the use of McDonald’s *ω* for examination of the internal consistency and sex invariance.

Additionally, it is imperative that some limits be recognized and addressed in further research projects. The primary drawback lies in the fact that generalizability of conclusions cannot be made due to various reasons, including (1) the sole recruitment of a non-clinical sample that consisted of adults residing in and are citizens of Lebanon, and (2) the predominance of females (66.5%) in the sample. The sample was obtained using the snowball sampling, a method in which initial participants refer to others creating a growing chain until the required sample size is reached or novel information stops emerging. This method’s lack of randomness is often criticized as it can limit the sample’s representativeness (Parker and Scott, 2019). In addition, using Google Forms excludes individuals without internet access or usage, thereby introducing a potential selection bias. For instance, a predominance of female or individuals from a single demographic group may be noticed among the sample. In fact, as suggested by Noy (2008), women may be overrepresented in snowball sampling as they are more likely to cooperate. Mental health disorders were identified using self-reported physician diagnosis, which may be subject to recall bias, misclassification or underreporting; this may potentially introduce bias into the study findings. Also, the present study abstained from investigating some psychometric characteristics of the Arabic ALS-18, such as test–retest reliability, which assesses the consistency of a measure when administered to the same individual on different occasions (Vilagut, 2024), calling for more research in these areas. A reliable measure should yield stable results over time, ensuring the scale’s validity by verifying that score differences reflect actual variations rather than random errors (Vilagut, 2024). In mental health research, this psychometric property is valuable for it assesses the effectiveness of intervention and treatment (Drinkwater et al., 2023). Reliability coefficients are very high (*ω* = 0.96 for the total score), which may indicate item redundancy. Finally, future validation studies still need to verify that the Arabic ALS-18 is valid, reliable and suitable for use among other Arabic-speaking cultures other Arabic-speaking cultures (such as Gulf, North African, Levantine populations) and clinical populations (such as patients with bipolar disorders, depressive disorders or personality disorders). The latter would allow for clinical validation and applicability of the results to diverse populations, possibly determining any implications for intervention or clinical screening (Schwarzer et al., 2022).

## 5 Conclusion

The current study sought to investigate the psychometric properties of the ALS-18 within an Arabic-speaking population, providing evidence of scale’s adequacy for assessing affective lability in Arab settings, particularly among a sample of Arabic-speaking adults residents and citizens of Lebanon. Additional cross-cultural validations of the ALS-18 in various cultural and religious contexts across Arab countries is suggested. Providing scholars access to the Arabic version of the scale may expand study prospects in this topic across various Arab cultural backgrounds and contexts.

## Data Availability

The original contributions presented in the study are included in the article/supplementary material, further inquiries can be directed to the corresponding author/s.
